# Nonadherence Factors and Sociodemographic Characteristics of HIV-Infected Adults Receiving Antiretroviral Therapy in Nnamdi Azikiwe University Teaching Hospital, Nnewi, Nigeria

**DOI:** 10.1155/2013/843794

**Published:** 2013-11-28

**Authors:** Ijeoma Okoronkwo, Uchenna Okeke, Anthonia Chinweuba, Peace Iheanacho

**Affiliations:** ^1^Department of Nursing Sciences, University of Nigeria, Enugu Campus, Enugu, Nigeria; ^2^Nursing Services Department, Nnamdi Azikiwe University Teaching Hospital, Nnewi, Nigeria

## Abstract

Adherence to treatment instructions with antiretroviral therapy (ART) is very crucial for successful treatment outcome. However, sticking to treatment instructions pose-great challenges to HIV/AIDS patients. This cross-sectional study was on HIV infected adults attending ART clinic in Nigeria to explore nonadherence factors in relation to their socioeconomic characteristics. Validated structured questionnaire was administered to 221 participants. Results showed a high nonadherence rate of 85.1%. The commonest occurring factors of non-adherence were forgetfulness (53.8%), busy schedule (38.8%), side effects of drugs (31.9%), and stigma (31.9%). Males were more likely to complain from busy schedule, feeling healthy, fear of partner disclosure, long waiting period, and long term regimen. Patients with no formal education were more likely to attribute non-adherence to poor communication, side effects of drugs, and stigma. Employed patients seemed to miss their drugs more than the unemployed and artisans. The high non-adherence rate has serious implications for the control of HIV in infected individuals and management of HIV in general. Nurses should intensify efforts on patient education and counseling.

## 1. Introduction

HIV/AIDS is one of the major public-health problems worldwide, affecting mostly people who are at the most productive stage of life. Out of the estimated 34 million people living with HIV/AIDS globally as at the end of 2010, 68% reside in Sub-Saharan Africa [1]. Nigeria has an HIV prevalence of 4.1% and currently an estimated 3.6 million people are living with HIV/AIDS [2] which puts Nigeria as the second country with the largest number of people living with HIV (PLWH) after South Africa.

The development and availability of antiretroviral therapy (ART) became a turning point in the control and prevention of the epidemic. With the success of ART in improving the quality of life of PLWH and reducing morbidity and mortality, HIV has become a chronic manageable disease [3]. Studies have shown a correlation between higher levels of adherence and improved virological and clinical outcomes [4, 5]. However, ensuring that PLWH receive, and adheres to their highly active ART poses a challenge to the treatment efficacy [6].

In Nigeria ART was commenced (i.e., combining at least 3 drugs from various classes of ART into a cocktail that produces a dramatic reduction in viral load and allows immune suppression) in 2002 and coverage stood at 359,181 in 2010 [2]. Adherence to ART according to Carter [7] refers to timely intake of the right dose of prescribed pills through the right route while observing dietary restrictions. Some available methods for measuring adherence have been reported to include self-reports (through interviews, surveys, and diaries), pill counts, clinical assessments, prescription refills, and directly observed therapy (DOT) [8, 9]. Getting clients to take drugs daily is one of the greatest challenges for effective ART as missing few doses can lead to drug resistance. World Health Organization [10] observes that patients with long term illness have problems with adhering to treatment instructions. It therefore becomes pertinent that treatment instructions are strictly adhered to in order to optimize ART effectiveness.

In Nigeria, there are designated health facilities that run daily retroviral clinics as well as provide free ART. This study was conducted in one of such facilities where PLWH are treated principally with two groups of drugs classified as “first line” and “second line”. The first line, which is the focus of this study, is presented as “combipak” containing three drugs—Zidovudine + Lamivudine + Nevirapine. The combination is administered one tablet each, twice daily. Patients are followed up on monthly appointments. In line with WHO's [11] revised ART guideline for resource constrained settings, highly active antiretroviral therapy is initiated on all patients with CD4 + T cell count below 350 cells/*μ*L, regardless of symptom. On each appointment, CD4 + T cell count is repeated and adherence to drug is ascertained by pill count of the previous supply. Effort is made through counseling to retain each client on the first line drugs due to their benefits because the second line drugs have more side effects. Various health care providers—including clinical psychologists—are involved in client counseling to encourage adherence.

However, it is often observed that some of these patients still fail to adhere to their treatment regimen. These patients return to the clinic for followup looking very ill with their drugs supply unexhausted. Various authors have observed that many PLWH do not adhere strictly to their treatment regimen [8, 9, 12], which according to Carter [7] might involve missing a drug in a combined therapy or a dose of the combined therapy, improper medication timing, and/or not taking the correct dose of any of the drugs.

Various factors have been identified as posing challenges to adherence such as forgetfulness, side effects of the drugs, illiteracy, and stigmatization [12–14]. In addition, Agu et al. [15] and Bello [16] identified high pill burden, ART-associated and travel costs, being female, single, high educational status, lack of knowledge, negative perception towards ART medication, and nonavailability of drugs at treatment site as other possible factors. There is paucity of research on how these factors become manifested on a population with diverse sociodemographic characteristics. This study investigated factors affecting non-adherence to ART among adult HIV-infected patients attending the ARV clinic of a hospital in southeast Nigeria and related these to their sociodemographic characteristics.

## 2. Methods

### 2.1. Research Design and Study Participants

A cross-sectional descriptive survey was conducted at the adult retroviral clinic of the Nnamdi Azikiwe University Teaching Hospital, Nnewi, southeast Nigeria. This centre is one of the designated free HIV retroviral outpatient clinics in the country. Two hundred and twenty-one adult HIV-infected patients were consecutively recruited into the study based on the inclusion criteria of: adult patients willing to participate and those who have been on ART for at least 3 months. Each patient that attended the ART outpatient clinic and who met the inclusion criteria was approached by one of the researchers; the purpose of the study, his/her role in the study, and the implication of his/her involvement were explained before a signed consent to participate was obtained.

The minimum sample size of 221 was determined using the statistical formula of Fisher for calculating sample size: *Nf* = *N*/1 + (*N*)/(*n*), where *N* = *Z* ^2^ *pq*/*d* ^2^ and *n* = estimated study population [17]. Average monthly attendance of HIV-infected adults from hospital records was 1,680.

### 2.2. Data Collection

The instrument used for this study was ART non-adherence and socio-demographic characteristics questionnaire (ARTNa-SDCQ) developed by the researchers based on the literature review. The questionnaire contained two sections. Section A was on sociodemographic data of respondents; section B contained four close-ended questions on: the duration of time a patient had been taking drugs; whether; ever missed drugs; how often drugs were missed and reasons for non-adherence (for those who admitted ever missing drugs, with eighteen possible options). Adherence was defined in this study as having taken at least 95% of the total prescribed pills at the prescribed dosing intervals as recommended by WHO [10]. This was determined by left-over pill count and self-report. Respondents were required to select as many options as they felt were contributory to not taking their drugs as prescribed. The factors were grouped as client, institutional, and medication related.

The reliability of the instrument was established by pilot testing it in an outstation ART clinic similar in characteristics to the study population. Twenty adult HIV-infected patients attending the ART clinic who met the study inclusion criteria were used. Data obtained were subjected to Cronbach's alpha test. An alpha of 0.72 and a standardized item (inter item) coefficient of 0.85 were obtained.

### 2.3. Ethical Considerations

The study was approved by the Research Ethical Committee of Nnamdi Azikiwe University Teaching Hospital, Nnewi, where the study was to be conducted. Permission was obtained from the management of the ARV clinic. Subsequently, each prospective respondent was approached by any of the researchers and the purpose and demands of the study were explained. They were informed that participation was voluntary and were assured of anonymity and confidentiality of information before informed consent was obtained.

Copies of the questionnaire were administered directly to the consenting patients and left-over pills were counted by the researcher to corroborate patient's response. Any patient with more than 5% left over (i.e., more than 3 pills) of each drug was considered nonadhering. Data were collected on each clinic day before the routine clinic activities commenced. This was to reduce patients' distractions in form of going for CD4 count check and other laboratory investigations. The researchers assisted those who needed help to complete the questionnaire. Data collection continued on subsequent clinic days until the sample size was reached and this lasted for one month.

### 2.4. Data Analysis

Data collected on demographic characteristics and adherence to drugs were analyzed descriptively. Responses to factors responsible for non-adherence were categorized into three groups: client, institutional, and medication related factors. The relationship between respondents' demographic characteristic, and their reasons for non-adherence to drugs were analyzed inferentially using GraphPad Prism 5.20 statistical package. *t*-test for unpaired data was used to analyze the relationship between respondents' adherence to ART and their demographic characteristics of sex and marital status, while analysis of variance (ANOVA) was employed to establish the relationship with their age, educational qualification, and employment status at *P* ≤ 0.05.

## 3. Results

### 3.1. Sociodemographic Characteristics

Of the 221 respondents, 188 (85.1%) indicated that they missed taking their drugs as prescribed in the preceding month while only 33 (14.9%) claimed they had never missed. Of the 188 patients that admitted non-adherence, 86 (45.7%) have been on ART for less than one year and 72 (38.3%) were on drugs between one year and 2.5 years, while the remaining 30 (16.0%) maintained the intake beyond 2.5 years and up to 5 years.

### 3.2. Factors of Nonadherence and Relationship with Respondents' Sex

Out of the 188 patients on ART who indicated they had missed taking their drugs as prescribed in the preceding month, 126 (67.0%) were females while 62 (33.0%) were males (see Table 1). The majority of 101 (53.7%) of the respondents, identified forgetfulness as a factor of non-adherence, the rate of which was almost the same for both sexes (females = 54%, and males = 53.2%). There was significant difference in the means of responses on the non-adherence factors identified by male and female patients on ART. At 95% CI and df = 34, the unpaired *t*_cal_ was 2.414 and *P* = 0.0213. The mean ± SEM of gender in relation to the factors was 25.22 ± 4.16 for the females and 14.39 ± 1.69 for the males. Except for forgetfulness, poor communication and side effects of drugs were identified by higher percentages of females than the males; more males than females identified the rest of the items.

### 3.3. Factors of Nonadherence and Relationship with Respondents' Age

There was no definite pattern in the responses based on age (see Table 2). However, forgetfulness (58.2%) and busy schedule (52.7%) were most frequent among respondents aged 40–49 years. Respondents aged 30–39 were nonadherent due to institutional factors of long waiting periods (44.7%) and poor communication (38.1%). Respondents aged 20–29 years (33.9%) were the highest in non-adherence because they simply “do not feel like taking drugs” and are “ignorant of the consequences” (23.7%). ANOVA results with df = 53 showed no significant difference in non-adherence factors among the groups (*F* = 2.440 and *P* = 0.0973).

### 3.4. Factors of Nonadherence and Relationship with Respondents' Marital Status

One hundred and seven (56.9%) of the respondents were married, while the remaining 81 (43.1%) were single (never married, divorced, or widowed). Many of the items had close responses from the groups (see Table 3). However, respondents who were single 31 (38.3%) were more nonadhering due to poor communication by care providers than the married patients 32 (29.9%). Similarly, the single respondents did not adhere to the ART regimen due to stigma 35 (43.2%) and long-term regimen of drug intake 25 (30.9%). Mean ± SEM of the reported non-adherence factors for both groups were similar (single = 19.00 ± 2.69; married = 20.61 ± 3.071). Unpaired *t*-test of the data at 95% CI and df = 34 showed *t*_cal_ as 0.3948 and *P* = 0.6955. Overall, there was no significant difference in non-adherence factors identified by the married and single adult HIV-infected patients on ART.

### 3.5. Factors of Nonadherence and Relationship with Respondents' Educational Status

The majority of the respondents had some formal education. Seventy-seven, 52, and 28 had secondary, higher and primary education, respectively, while 31 had none (see Table 4). All categories of the respondents attributed their non-adherence to forgetfulness. In addition, those that had higher education (55.8%) and those with no formal education (51.6%) identified busy schedule. Also, those with no formal education failed to adhere to their ART due to poor communication (61.3%), side effects of drugs (58.1%), stigma (54.8%), and lack of trust and confidentiality (38.7%). Results of ANOVA at df = 71 showed a significant difference in non-adherence factors and respondents' educational status (*F* = 4.839 and *P* = 0.0041).

### 3.6. Factors of Nonadherence and Relationship with Respondents' Employment Status

Employed respondents 92 (48.9%) were more likely to miss their drugs when compared with the artisans 95 (29.3%) and unemployed groups 41 (21.8%).  Table 5 shows that employed respondents identified, among others, forgetfulness 49 (53.3%), busy schedule 42 (45.6%), and side effects of drugs 38 (41.3%) as their factors of non-adherence. Artisans and the unemployed had lower scores for most of the items. Analysis of variance (ANOVA) of the data showed a significant difference in non-adherence factors and respondents employment status (df = 53; *F* = 9.525; *P* = 0.0003).

## 4. Discussion

The rate of nonadherence to antiretroviral drugs among patients receiving ART in Nnamdi Azikiwe University Teaching Hospital, Nnewi, as shown in this study was rather high as only 14.9% (33) of the subjects indicated that they have never missed their medication. This is particularly significant considering that the method of data collection was self-report. Although previous studies had noted that some PLWH do not adhere strictly to their treatment regimen [8, 9] the rate in the present study was rather much higher than what was reported in similar studies. For instance, Igwegbe et al. [8] had worked in the same locality, though among pregnant women and found about 21.7% being nonadherent. However, the findings of Uzochukwu et al., [12] which reported a non-adherence rate of 75%, were closer to that of the present study. The consequence of this level of non-adherence is worrisome both for the individual and the entire population because of the virological outcomes. A possible explanation for this finding is the fact that the method used was self-report. According to Uzochukwu et al., [12] a common limitation of this method in assessing adherence is its subjectivity. For example the patient may say what he feels that may help him get the desired attention from his doctor or what he thinks will impress the doctor.

### 4.1. Socio-Demographic Variables and Factors of Nonadherence

With regard to gender and factors of non-adherence, it was noticed that more males than females were more likely to complain of factors like busy schedule, fear of partner disclosure, long waiting period, ignorance of consequences, long term regimen, and stigma. However, more females than males seemed to be non-adherent due to forgetfulness, poor communication, and side effects of drugs. These findings are not quite in agreement with those of Wasti et al., [14] where women were generally found to be more nonadherent than men. Busy schedule and forgetfulness will likely work together to affect non-adherence. Ignorance of consequences of non-compliance is a high risk for non-adherence. Good communication promotes access to care and adherence to ART. With effective communication, patients must be made aware that ART is a lifelong treatment [9]. This suggests the need for adequate client communication and assessment of understanding. Health care providers need to spend sufficient time in adherence counseling at every visit.

Concerning the relationship between non-adherence factors and age, the 40–49 years age group was the most nonadherent due to forgetfulness. Social responsibilities tend to increase with age and this may probably have been the case with this e eldest group in this study. Patients aged 20–29 years were most frequently nonadherent because they did not feel like taking drugs. The findings also interestingly showed that the same group reported ignorance of the consequences of their non-adherence more than the other two groups. However, statistical analysis did not show any significant variation in the factors of non-adherence as a result of age.

There was also no significant difference in non-adherence factors between the married and the single respondents. This finding is not consistent with that of Agu et al. [15] and Bello [16]. Nevertheless single patients seemed to be more non-adhering due to poor communication by care providers than the married. Similarly, they were also likely to be more nonadhering due to stigma and long term regimen. This finding could be explained by the extent of social support the patient has. Married clients, especially those whose spouses are either seropositive or are aware of their HIV status, are likely to have people who support and encourage them and this can enhance adherence on the long term as well as reduce stigma.

Educational status had a significant influence on some of the factors associated with non-adherence although no definite pattern was shown. Forgetfulness was common to all the categories. However, those with higher education were more likely than the other groups to attribute their non-adherence to busy schedule. Patients with no formal education were more likely to attribute non-adherence to poor communication, side effects of drugs, stigma, and lack of trust and confidentiality. Although Agu et al. [15] and Bello [16] had earlier identified higher educational status as a factor of non-adherence, the findings of our study did not show any correlation of educational status with non-adherence. However, education obviously plays a considerable role in understanding and communication of information between parties.

The results further showed that employed patients were more likely to cite forgetfulness, busy schedule, and stigma as reasons for non-adherence than the unemployed and artisans. This finding was statistically significant. The possible explanation is that their jobs kept them busy making them forget their drugs. Also the fact of working in an employed job may heighten their fear of stigma since they spend a good chunk of their time in the company of other people in their places of work; this may contribute to their forgetfulness. Stigma will not make them bring out their drugs in the office. Fear of stigma in the place of work may also be due to fear of losing their jobs if their HIV statuses are known.

On the basis of the foregoing, in-depth one-on-one counseling will most likely help clients to resiliently withstand sigma, accept their condition, and adapt more positively to the challenges of the illness and prolonged drug use. Again, follow-up of patients on ART will most likely help to reduce factors of non adherence like forgetfulness, ignorance, and lack of confidence in treatment.

## 5. Conclusion

Non-adherence was found to be high among the respondents in this study and several factors were cited as reasons. More information, education, and communication campaigns targeted at the general public are required to eliminate or reduce stigma to the barest minimum. This will help to improve adherence for those who might have the need to take their medications outside their homes, such as those in paid employments. Policies should be made and rules should be enforced to protect the rights of PLWH in the work place as well as prevent any form of discrimination against them. Health care providers need to spend sufficient time in adherence counseling at every visit emphasizing eating before drugs intake to minimize side effects. Strategies to automatically remind patients at due medication times such as setting alarms will be invaluable to reduce forgetfulness. Further studies are required to determine particular drugs which clients find most problematic.

## Figures and Tables

**Table 1:** Factors of nonadherence and relationship with respondents' sex.

Reasons for nonadherence	*n* (%)	Female *n *= 126 (67%)	Male *n *= 62 (33%)
Personal factors			
Forgetfulness	101 (66)	68 (54.0)	33 (53.2)
Busy schedule	73 (47.7)	47 (37.3)	26 (41.9)
Feeling healthy	30 (16.0)	20 (15.9)	10 (16.1)
Ignorance of consequences	33 (21.6)	19 (15.1)	14 (22.6)
Poverty	12 (6.4)	7 (5.6)	5 (8.1)
Do not feel like taking drugs	38 (24.8)	25 (19.8)	13 (21.0)
No confidence in the treatment	17 (9.0)	9 (7.1)	8 (12.9)
Fear of partner disclosure	29 (15.4)	16 (12.7)	13 (21.0)
Provider/institutional factors			
Poor communication	63 (41.2)	49 (38.9)	14 (22.6)
Judgmental attitude of provider	20 (13.1)	11 (8.7)	9 (14.5)
Lack of trust and confidentiality	42 (27.5)	25 (19.8)	17 (27.4)
Long waiting period	38 (24.8)	20 (15.9)	18 (29.0)
Unavailability of drugs	13 (6.9)	6 (4.8)	7 (11.3)
Drug related factors			
Side effects of drugs	60 (39.2)	48 (38.1)	12 (19.4)
Stigma	60 (17.7)	38 (30.2)	22 (35.5)
Frequency of taking drugs	27 (39.2)	14 (11.1)	13 (21.0)
Long term regimen	41 (26.8)	23 (18.3)	18 (29.0)
Quantity of drugs to be taken	16 (8.5)	9 (7.1)	7 (11.3)
Mean ± SEM of sex		25.22 ± 4.16	14.39 ± 1.69
Unpaired *t*-test result			
t_cal_		2.414	
Df		34	
95% confidence interval		1.708 to 19.96	
*P* value		0.0213*	
Are means significantly different? (*P* < 0.05)		Yes	

*Note.* Percentage was calculated based on proportion of nonadherent groups.

*The scores are statistically significantly different (*P* < 0.05).

**Table 2:** Factors of nonadherence and relationship with respondents' age.

Reasons for nonadherence	*n* (%)	20–29 *n* = 59 (31.4%)	30–39 *n* = 74 (36.4%)	40–49 *n* = 55 (29.2%)
Personal factors				
Forgetfulness	101 (53.7)	27 (45.8)	42 (56.8)	32 (58.2)
Busy schedule	73 (38.8)	16 (27.1)	28 (37.8)	29 (52.7)
Feeling healthy	30 (15.9)	1 (1.6)	15 (20.3)	14 (25.4)
Ignorance of consequences	33 (17.5)	14 (23.7)	13 (17.6)	6 (10.9)
Poverty	12 (6.4)	4 (6.8)	6 (8.1)	2 (3.6)
Do not feel like taking drugs	38 (20.2)	20 (33.9)	16 (21.6)	2 (3.6)
No confidence in the treatment	17 (9.0)	4 (23.7)	9 (12.2)	4 (7.2)
Fear of partner disclosure	29 (15.4)	7 (11.9)	18 (24.3)	4 (7.2)
Provider/institutional factors				
Poor communication	63 (33.5)	22 (37.3)	24 (38.1)	17 (30.9)
Judgmental attitude of provider	20 (10.6)	8 (13.6)	8 (32.4)	4 (7.2)
Lack of trust and confidentiality	42 (22.3)	14 (23.7)	20 (27.0)	8 (14.5)
Long waiting period	38 (20.2)	15 (25.4)	17 (44.7)	6 (10.9)
Unavailability of drugs	13 (6.9)	4 (6.8)	5 (23.0)	4 (7.2)
Drug related factors				
Side effects of drugs	60 (31.9)	18 (30.5)	21 (28.4)	21 (38.2)
Stigma	60 (31.9)	20 (33.9)	23 (31.1)	17 (30.9)
Frequency of taking drugs	27 (14.36)	14 (23.7)	10 (13.5)	3 (5.4)
Long term regimen	41 (21.8)	19 (32.2)	15 (20.3)	7 (12.7)
Quantity of drugs to be taken	16 (8.5)	9 (15.2)	6 (8.1)	1 (1.8)
ANOVA summary result				
*F*		2.440		
Df		2		
*P* value		0.0973		
Are means significantly different? (*P* < 0.05)		No		

**Table 3:** Factors of nonadherence and relationship with respondents' marital status.

Reasons for nonadherence	*n* (%)	Single *n = *81 (43.1%)	Married *n = *107 (56.9%)
Personal factors			
Forgetfulness	101 (53.7)	43 (51.9)	58 (54.2)
Busy schedule	73 (38.8)	32 (39.5)	41 (38.3)
Feeling healthy	30 (15.9)	14 (17.3)	16 (15.0)
Ignorance of consequences	33 (17.5)	17 (21.0)	16 (15.0)
Poverty	12 (6.4)	5 (6.2)	7 (6.5)
Do not feel like taking drugs	38 (20.2)	17 (21.0)	21 (19.6)
No confidence in the treatment	17 (9.0)	7 (8.6)	10 (9.4)
Fear of partner disclosure	29 (15.4)	10 (12.4)	19 (17.8)
Provider/institutional factors			
Poor communication	63 (33.5)	31 (38.3)	32 (29.9)
Judgmental attitude of provider	20 (10.6)	10 (12.4)	10 (9.4)
Lack of trust and confidentiality	42 (22.3)	19 (23.5)	23 (21.5)
Long waiting period	38 (20.2)	20 (24.7)	18 (16.8)
Unavailability of drugs	13 (6.9)	6 (7.4)	7 (6.5)
Drug related factors			
Side effects of drugs	60 (31.9)	31 (38.3)	29 (27.1)
Stigma	60 (31.9)	35 (43.2)	25 (23.4)
Frequency of taking drugs	27 (14.36)	13 (16.1)	14 (13.1)
Long term regimen	41 (21.8)	25 (30.9)	16 (15.0)
Quantity of drugs to be taken	16 (8.5)	7 (8.6)	9 (8.4)
Mean ± SEM of marital status		19.00 ± 2.69	20.61 ± 3.07
Unpaired *t*-test result			
*t*_cal_		0.3948	
Df		34	
95% confidence interval		−9.910 to 6.688	
*P* value		0.6955	
Are means significantly different? (*P* < 0.05)		No	

**Table 4:** Factors of nonadherence and relationship with respondents' educational status.

Reasons for nonadherence	*n* (%)	No formal *n* = 31 (16.5)	Primary *n* = 28 (14.9)	Secondary *n* = 77 (41.0)	Higher *n* = 52 (27.6)
Personal factors		20 (64.5)	15 (53.6)	36 (46.7)	30 (57.7)
Forgetfulness	101 (53.7)				
Busy schedule	73 (38.8)	16 (51.6)	10 (35.1)	18 (23.4)	29 (55.8)
Feeling healthy	30 (15.9)	6 (19.3)	4 (14.3)	12 (15.6)	8 (15.4)
Ignorance of consequences	33 (17.5)	7 (22.6)	5 (17.9)	17 (22.1)	4 (7.7)
Poverty	12 (6.4)	3 (9.7)	2 (7.1)	4 (5.2)	3 (5.8)
Do not feel like taking drugs	38 (20.2)	8 (25.8)	6 (21.4)	15 (19.5)	9 (17.3)
No confidence in the treatment	17 (9.0)	4 (12.9)	3 (10.7)	6 (7.8)	4 (7.7)
Fear of partner disclosure	29 (15.4)	6 (19.3)	4 (14.3)	13 (16.9)	6 (11.5)
Provider/institutional factors					
Poor communication	63 (33.5)	19 (61.3)	5 (17.9)	20 (26.0)	19 (36.5)
Judgmental attitude of provider	20 (10.6)	4 (12.9)	2 (7.1)	7 (9.1)	7 (13.5)
Lack of trust and confidentiality	42 (22.3)	12 (38.7)	7 (25.0)	12 (15.6)	11 (21.1)
Long waiting period	38 (20.2)	6 (19.3)	7 (25.0)	14 (18.2)	11 (21.1)
Unavailability of drugs	13 (6.9)	3 (9.7)	2 (7.1)	5 (6.5)	3 (5.8)
Drug related factors					
Side effects of drugs	60 (31.9)	18 (58.1)	4 (14.3)	16 (20.8)	22 (42.3)
Stigma	60 (31.9)	17 (54.8)	9 (32.1)	18 (23.4)	16 (30.8)
Frequency of taking drugs	27 (14.36)	5 (16.1)	2 (7.1)	10 (13.0)	10 (19.2)
Long term regimen	41 (21.8)	8 (25.8)	8 (28.6)	15 (19.5)	10 (19.2)
Quantity of drugs to be taken	16 (8.5)	4 (12.9)	3 (10.7)	5 (6.5)	4 (7.7)
ANOVA summary result					
*F *		4.839			
Df		3			
*P* value		0.0041**			

**The scores are statistically significantly different (*P* < 0.05).

**Table 5:** Factors of nonadherence and relationship with respondents' employment status.

Reasons for nonadherence	*n* (%)	Employed *n* = 92 (48.9)	Unemployed *n* = 41 (21.8)	Artisan *n* = 55 (29.3)
Personal factors				
Forgetfulness	101 (53.7)	49 (53.3)	23 (56.1)	29 (52.7)
Busy schedule	73 (38.8)	42 (45.6)	14 (34.1)	17 (30.9)
Feeling healthy	30 (15.9)	13 (14.1)	8 (19.5)	9 (16.4)
Ignorance of consequences	33 (17.5)	18 (19.6)	6 (14.6)	9 (16.4)
Poverty	12 (6.4)	7 (7.6)	3 (7.3)	2 (3.6)
Do not feel like taking drugs	38 (20.2)	17 (18.5)	10 (24.4)	11 (20.0)
No confidence in the treatment	17 (9.0)	10 (10.9)	4 (9.8)	3 (5.4)
Fear of partner disclosure	29 (15.4)	13 (14.1)	9 (21.9)	7 (12.7)
Provider/institutional factors				
Poor communication	63 (33.5)	31 (33.7)	22 (53.7)	10 (18.2)
Judgmental attitude of provider	20 (10.6)	9 (9.8)	6 (14.6)	5 (9.1)
Lack of trust and confidentiality	42 (22.3)	23 (25.0)	7 (17.1)	12 (21.8)
Long waiting period	38 (20.2)	17 (18.5)	11 (26.8)	10 (18.2)
Unavailability of drugs	13 (6.9)	6 (6.5)	4 (9.8)	3 (5.4)
Drug related factors				
Side effects of drugs	60 (31.9)	38 (41.3)	13 (21.7)	9 (16.4)
Stigma	60 (31.9)	31 (33.7)	14 (34.1)	15 (27.3)
Frequency of taking drugs	27 (14.36)	14 (15.2)	7 (17.1)	6 (10.9)
Long term regimen	41 (21.8)	26 (28.3)	4 (9.8)	11 (20.0)
Quantity of drugs to be taken	16 (8.5)	9 (9.8)	4 (9.8)	3 (5.4)
ANOVA summary result				
*F *		9.525		
Df		2		
*P* value		0.0003***		

***The scores are statistically significantly different (*P* < 0.05).

## References

[1] UNAIDS Joint United Nations Programme on HIV/AIDS (UNAIDS) World AIDS Day report.

[2] National Agency for the control of AIDS (NACA) Update on the HIV/AIDS epidemic response in Nigeria. http://www.naca.gov.ng/index2.php.

[3] Oguntibeju OO (2012). Quality of life of people living with HIV and AIDS and antiretroviral therapy. *HIV/AIDS-Research and Palliative Care*.

[4] Harrigan PR, Hogg RS, Dong WWY (2005). Predictors of HIV drug-resistance mutations in a large antiretroviral-naive cohort initiating triple antiretroviral therapy. *Journal of Infectious Diseases*.

[5] Orrell C, Bangsberg DR, Badri M, Wood R (2003). Adherence is not a barrier to successful antiretroviral therapy in South Africa. *AIDS*.

[6] Granich RM, Gilks CF, Dye C, De Cock KM, Williams BG (2009). Universal voluntary HIV testing with immediate antiretroviral therapy as a strategy for elimination of HIV transmission: a mathematical model. *The Lancet*.

[7] Carter M Adherence. Information series for HIV positive people. NAM. http://www.aidsmap.com/Adherence/cat/1464/.

[8] Igwegbe AO, Ugboaja JO, Nwajiaku LA (2010). Prevalence and determination of non adherence to antiretroviral therapy among HIV positive pregnant women in Nnewi, Nigeria. *International Journal of Medicine and Medical Sciences*.

[9] Nyambura AW (2009). *Factors that influence non-adherence to antiretroviral therapy among HIV/AIDS patients in central province, Kenya [Ph.D. thesis]*.

[10] World Health Organization (WHO) (2004). *Adherence to HIV Treatment*.

[11] World Health Organization (WHO) (2009). *Rapid Advice: Antiretroviral Therapy for HIV Infection in Adults and Adolescents*.

[12] Uzochukwu BSC, Onwujekwe OE, Onoka AC, Okoli C, Uguru NP, Chukwuogo OI (2009). Determinants of non-adherence to subsidized anti-retroviral treatment in southeast Nigeria. *Health Policy and Planning*.

[13] Monjok E, Smesny A, Okokon IB, Mgbere O, Essien EJ (2010). Adherence to antiretroviral therapy in Nigeria: an overview of research studies and implications for policy and practice. *HIV/AIDS*.

[14] Wasti SP, Simkhada P, Randall J, Freeman JV, van Teijlingen E (2012). Factors influencing adherence to antiretroviral treatment in Nepal: a mixed-methods study. *PLoS ONE*.

[15] Agu KA, Okojie O, King RG (2011). Medication adherence and risk factors for non-adherence among patients taking highlyactive retroviral therapy. *West African Journal of Pharmacy*.

[16] Bello SI (2011). HIV patients’adherence to antiretroviral therapy in Sobi Specialist Hospital, Ilorin, Nigeria. *Journal of Advanced Scientific Research*.

[17] Araoye MO (2003). *Research Methodology with Statistics for Health and Social Sciences*.

